# Partially systematic thoughts on the history of systematic reviews

**DOI:** 10.1186/s13643-018-0833-3

**Published:** 2018-10-27

**Authors:** Mike Clarke

**Affiliations:** Northern Ireland Methodology Hub, Centre for Public Health, Institute of Clinical Sciences, Block B, Queen’s University Belfast, Royal Hospitals, Grosvenor Road, Belfast, BT12 6BJ Northern Ireland

## Abstract

Six years after the launch of *Systematic Reviews* by Biomed Central, this article is part of the celebration of the journal. It contains personal reflections on the past, present and future of systematic reviews, using examples relevant to the role of systematic reviews in cataloguing and analysing research, assessing quality and planning new studies. The focus is on the most common of the various types of systematic review in health and social care, namely those assessing the effects of interventions.

## Background

Shortly before the start of the twentieth century, George Gould presented a vision to the inaugural meeting of the Association of Medical Librarians in Philadelphia on May 2, 1898: “I look forward to such an organisation of the literary records of medicine that a puzzled worker in any part of the civilized world shall in an hour be able to gain a knowledge pertaining to a subject of the experience of every other man in the world” [1].

Now, 120 years on, 114 years since Karl Pearson published a pooled analysis of the results of a series of studies to investigate the effects of a typhoid vaccine [2], 42 years since Gene Glass coined the term “meta-analysis” [3], 25 years since the Cochrane Collaboration (now, simply, Cochrane) was established, and 6 years since the journal *Systematic Reviews* began, I am pleased to be part of the journal’s celebration and to share my thoughts on the history of this type of research, which was recently described as entering a “midlife crisis” [4]. It is a history that the journal has helped to document, publishing research articles for many aspects of the methods for systematic reviews, which are likely to feature in historical accounts now and far into the future.

It is, of course, strange to be writing about the history of something that is living, growing and changing, and of which I feel very much part. However, finding myself increasingly using the phrase “years ago” when talking about projects I have been involved in, some of what has happened in not just the “distant” past is feeling historical, as I reflected in an essay for the James Lind Library [5].

Many people have said that history belongs to the victors and to some extent that is the case when we look back at what have become known as “systematic reviews” over the last century and more. But, I suggest that there is also an important role for the persistent, for those who have stuck at the task despite the challenges. For people involved in systematic reviews, recent decades have seen some of these challenges be overcome. Many tasks are now much easier for people preparing and using systematic reviews than even 10 years ago because of the persistence of individuals and organisations who have raised the profile, importance and value of systematic reviews. Initiatives that are now evolving and developing have come from that persistence and are likely to be seen as pivotal to those writing historical pieces in years and decades to come. I look at some of these areas in this article, helped by other accounts of the history of evidence synthesis [4–8].

I have not attempted to conduct my own “systematic review” of the history. Instead, I illustrate this history with examples relevant to the past, present and future, anchored around some of the key reasons for doing systematic reviews today. My main attention is on the role of systematic reviews in cataloguing and analysing research, but I also touch on their importance for assessing quality and planning new studies. I focus on the commonest type of systematic review in health and social care, those that assess the effects of interventions. Although many of the principles for doing these reviews have remained constant over time, their methods are evolving and a historian even just a few years from now may be looking back on a different landscape. They may be reflecting on even greater growth in the number of reviews that have been conducted as “rapid reviews” become more common as a means to quickly meet the specific needs of decision makers who do not wish to wait for a full systematic review. Rapid reviews speed up the processes for searching, appraisal, extraction and gathering of data extraction, and the analysis and synthesis of the findings of the included studies [9, 10]. And, in a further development, the concept of “living systematic reviews” has been introduced to refer to reviews where the updating process is almost continuous, adding new studies as soon as possible after they become available [11]. These accelerations may lead to bias, and analyses of their advantages and disadvantages compared to full reviews are likely to either confirm that things can be done quicker without compromising quality or instil caution in those users who feel that they cannot wait for a full review.

In recognition of how I am writing about a history that is itself “living”, and how I expect I have failed to refer to some key people, papers and developments, I welcome comment, feedback and debate on how my reflections match those of others who were, are and will be part of the past, present and future of systematic reviews.

## Cataloguing

One of the challenges facing anyone wishing to use the enormous amount of research that has been conducted in health and social care, and the dozens or, sometimes even, hundreds of individual studies addressing the same issue is finding this material. In 1994, Cindy Mulrow estimated that more than two million articles were being published in biomedicine every year and drew a mental picture of a tower 500 m high if all the journals were piled on top of each other [12]. Since then, the arrival and proliferation of online-only journals means that we might no longer share the concept of piles of print journals towering into the sky or occupying kilometres of library shelves, and have moved on to thinking about the number of terabytes of storage needed to cope with all of these articles. However, the growth in the number of articles has accelerated and many more than two million articles are now published every year. Thinking only of research that evaluates the effects of health and social care interventions, tens of thousands of controlled trials are published annually and more than 120,000 trials are currently open to recruitment according to a search of the World Health Organization’s International Clinical Trials Registry Platform [13] in August 2018 (apps.who.int/trialsearch).

Systematic reviews help users to find their way through this morass to the studies that they are most interested in, and global efforts to facilitate systematic reviews have greatly facilitated access to the individual studies over the last few decades. In the 1970s, Archie Cochrane wrote “It is surely a great criticism of our profession that we have not organised a critical summary, by speciality or subspeciality, adapted periodically, of all relevant randomised controlled trials” [14]. And, when setting out one of the challenges to be overcome by The Cochrane Collaboration in a *British Medical Journal* editorial in 1992, Iain Chalmers, Kay Dickersin and Tom Chalmers wrote “failing to conduct systematic, up to date reviews of controlled trials of health care may result in substantial adverse consequences for patients, practitioners, the health services, researchers, and research funding bodies” [15].

Of course, individual examples of such catalogues of critical summaries of research exist from well before the late twentieth century, but these were focused on specific topics. Most notably perhaps, James Lind’s treatise on scurvy in the eighteenth century included not only his own experiments on ways to prevent this disease but also his “critical and chronological view of what has been published on the subject” [16].

Systematic reviews today provide a valuable resource as a catalogue of the studies addressing the specific question for the review, but even without the reviews themselves, much has been done to bring those studies together. When The Cochrane Collaboration was established in 1993, approximately 20,000 reports of randomised trials could be easily found in the bibliographic database, MEDLINE. Through improved indexing and initiatives such as the MEDLINE retagging projects of the US and UK Cochrane Centres [17], this has now increased to more than 460,000 PubMed records tagged with the publication type for “randomized controlled trial” in August 2018. Nearly 60,000 of these were published before 1992 and can now be found with this simple search. The Cochrane Central Register of Controlled Trials itself now contains more than one million records, and prospective registries of trials which were called for in 1986 by John Simes [18] allow users to find both ongoing studies and many more that have closed. However, the findings of many of these studies have not been published [19], highlighting how this problem of selective reporting, called “scientific misconduct” by Iain Chalmers in 1990 [20], is ongoing.

Systematic reviews themselves are now in need of similar cataloguing exercises. Reviews are more readily retrievable in bibliographic databases than they were a few decades ago, but are increasing rapidly in number. Estimates for published reviews have risen from a total of about 3000 in MEDLINE for the whole of the period from 1980 to 2000 [21] to approximately 2500 per year in 2007 [22], through 4000 for 2010 [23] and towards 8000 for 2014 [24]. An August 2018 search of PubMed for records tagged with the publication type meta-analysis retrieved more than 11,000 records for publications from 2017 in that database alone, and Jessica Gurevitch et al. have estimated that the total available in the literature is already beyond 200,000 [4].

There are also collections of systematic reviews produced to a common standard, with an early exemplar being the dozens of systematic reviews of controlled trials relevant to maternity care brought together in the 1980s in *Effective Care in Pregnancy and Childbirth* [25], which evolved into The Cochrane Collaboration Pregnancy and Childbirth Database [7]. Today, we have the output produced by organisations such as Cochrane (www.CochraneLibrary.com), Campbell (www.campbellcollaboration.org/library.html) and the Joanna Briggs Institute JBI (lww.com/jbisrir/Pages/default.aspx).

There are also aggregators of systematic reviews, including the now archived Database of Abstracts of Reviews of Effects (DARE) for the effects of healthcare interventions generally (www.crd.york.ac.uk/CRDWeb), and smaller collections such as that produced by Evidence Aid to improve access to reviews relevant to the humanitarian sector (www.EvidenceAid.org) [26]. There is even a dedicated prospective register for systematic reviews, PROSPERO. In fact, the second article in *Systematic Reviews* in February 2012 presented the “nuts and bolts” of this new register [27]. A total of 200 reviews were registered in PROSPERO’s first 8 months, and it was warmly welcomed by Sally Davies, Director of the UK’s National Institute for Health Research [28]. By August 2018, nearly 40,000 ongoing reviews had been registered, growing by more than 10,000 per year, and methodology research is beginning to appear which uses these records [29, 30]. Finally, individual reviews themselves are now being subject to combination in overviews bringing together the findings of multiple reviews [31] and greater automation in the review process looks set to accelerate further both the number of reviews and the speed with which they are done [32–34].

## Analysing

In 1885, Lord Rayleigh told the meeting of the British Association for the Advancement of Science: “If, as is sometimes supposed, science consisted in nothing but the laborious accumulation of facts, it would soon come to a standstill, crushed, as it were, under its own weight. The suggestion of a new idea, or the detection of a law, supersedes much that has previously been a burden on the memory, and by introducing order and coherence facilitates the retention of the remainder in an available form. Two processes are thus at work side by side, the reception of new material and the digestion and assimilation of the old. One remark, however, should be made. The work which deserves, but I am afraid does not always receive, the most credit is that in which discovery and explanation go hand in hand, in which not only are new facts presented, but their relation to old ones is pointed out” [35].

When this combination of the old and the new is achieved using quantitative methods, we enter the realm of meta-analysis, the statistical combination of the results of related studies. In their recent account of the history of meta-analysis, which has a particular focus on its use over the last three decades in ecology, evolutionary biology and conservation, Gurevitch et al. described two different goals for those doing meta-analyses: a specific one of assessing the evidence for the effects of specific interventions or exposures for a particular problem or a more comprehensive one that seeks broad generalisations across large numbers of study outcomes [4].

The term meta-analysis was coined by Gene Glass in the mid-1970s and used in his American Educational Research Association presidential address in April 1976, describing it as “the analysis of analyses” [3]. The following year, with Mary Lee Smith, Glass published one of the largest meta-analyses, using data from 375 studies of psychotherapy and counselling, with a total of more than 25,000 people [36], providing an example of the type of comprehensive approach described by Gurevitch et al. [4]. Around the same time, pivotal papers on the statistical methods for combining the results of studies to assess the more specific effects they describe, were being published [37, 38]. Since then, as the statistical methods have developed, so have the means for displaying the results, with the introduction of the forest plot in the 1980s [39–41].

Looking to the future, some of the more advanced statistical techniques seem set to become more common. These include methods such as the re-analysis of the individual participant data from each included study [42, 43], which was used as far back as 1970 [44] and is likely to be boosted by greater access to the data from trials [45, 46], while the more recent introduction of the mixed treatment comparison or network meta-analyses approach [47] has seen a large rise in the number of published systematic reviews using this technique, with several hundred now available [48–51].

## Assessing quality and designing new studies

In 1753, Lind introduced his cataloguing of studies of scurvy and the need for their appraisal with “As it is no easy matter to root out prejudices,….it became requisite to exhibit a full and impartial view of what had hitherto been published on the scurvy … Indeed, before the subject could be set in a clear and proper light, it was necessary to remove a great deal of rubbish” [16]. Unfortunately, to this day, systematic reviews continue to face the problem that much of the existing research is “rubbish” or non-existent. A large proportion of Cochrane Reviews conclude that there is insufficient reliable evidence to determine whether there are important differences in the effects of the interventions they seek to compare.

The last couple of decades have seen historical developments in the assessment of the quality of existing research, leading to greater transparency in the process but also greater concerns about the amount of research waste due to poor-quality studies [52]. The Cochrane Risk of Bias tools for randomised [53] and non-randomised trials [54] allow reviewers to show their users how they reached their decisions, while the work of GRADE and GRADE-CERQual provides the means to present both the quality of the evidence brought together in the review and the strength of its recommendations [55–57]. We are also equipped to assess the quality of systematic reviews, developing from the original Oxman and Guyatt checklist [58] to two incarnations of AMSTAR [59, 60] for systematic reviews generally and checklists for specific types of review such as that from ISPOR for network meta-analyses [61]. There is also the QUOROM [62], now PRISMA guidance for reporting reviews [63], with extensions for reviews using individual participant data [64] and network meta-analysis [65]. However, application of these tools reveals that much work needs to be done to improve the quality of many systematic reviews [66, 67].

Given how many systematic reviews reach conclusions that reflect the inability of the existing evidence base to answer their research question and highlight the need for ongoing uncertainty about the relative effects of the interventions they investigated, one of the important benefits for systematic reviews is in providing the justification for the new studies that will fill this gap. Some of the early examples of systematic reviews recognised this. In 1976, Shaikh et al. reviewed 29 studies of tonsillectomy and adenoidectomy in children that had been published over the preceding 50 years and concluded by calling for a new, high-quality randomised trial that would ensure that “the methodologic pitfalls annotated in our review be guarded against” [68]. Reading the implications for research in more recent systematic reviews, one would see many similar calls [69]. Drawing on the past while looking to the future, these new studies need to be designed in the light of the existing evidence [70], report their findings in the context of the updated evidence [71, 72] and use methods that will minimise research waste [52, 73]. In doing so, these new studies will feed into future systematic reviews, and their conduct and that of the future systematic reviews will also be able to draw on another of the recent historical developments in systematic reviews: the systematic review of methodology [74]. The aforementioned rapid review process may also be of particular relevance here, as a means to quickly identify research gaps and uncertainties. “Rapid Research Needs Assessments” can highlight those areas most in need of a new study and the UK’s Public Health Rapid Support Team for disease outbreaks plans to work with Evidence Aid [26] to conduct such assessments in the early stages of a humanitarian emergency associated with a disease outbreak in order to identify important uncertainties that could be tackled by initiating new research.

## Conclusions

Returning to the nineteenth century quote from George Gould, with which I began this article, we now live in a world where technology means that people everywhere should be able to retrieve the knowledge they need about the effects of health and social care interventions in minutes, as long as barriers are not put in their way to access this [75]. If this knowledge is to be reliable and to have minimised bias, it will need to have been accumulated in systematic reviews. The last century and more, but particularly recent decades, have seen substantial developments to assist with this. Some of these can already be recognised as “historical”, others will become so in the years to come, and others will fade away. However, the ongoing persistence of those wishing to provide the evidence to help people making decisions and choices about their own health and social care, and that of others, will, I hope, ensure that systematic reviews will continue to have a future, as well as a past.
